# Acamprosate in a mouse model of fragile X syndrome: modulation of spontaneous cortical activity, ERK1/2 activation, locomotor behavior, and anxiety

**DOI:** 10.1186/s11689-017-9184-y

**Published:** 2017-06-12

**Authors:** Tori L. Schaefer, Matthew H. Davenport, Lindsay M. Grainger, Chandler K. Robinson, Anthony T. Earnheart, Melinda S. Stegman, Anna L. Lang, Amy A. Ashworth, Gemma Molinaro, Kimberly M. Huber, Craig A. Erickson

**Affiliations:** 1 Division of Psychiatry, MLC 7004, Cincinnati Children’s Research Foundation, 3333 Burnet Ave., Cincinnati, OH 45229-3039 USA; 2 Department of Neuroscience, University of Texas Southwestern Medical Center, Dallas, TX 75390 USA; 3 Present address: Division of Nephrology and Hypertension, Cincinnati Children’s Hospital Medical Center, Cincinnati, OH 45229 USA; 4 Present address: Department of Pharmacology and Toxicology, University of Louisville, Louisville, KY 40202 USA; 5 Present address: BlackbookHR, Cincinnati, OH 45202 USA

**Keywords:** Electrophysiology, Hyperactivity, Fragile X syndrome, Anxiety, Extracellular signal-related kinase, Dendritic spine density, Hippocampus, Striatum, Open field

## Abstract

**Background:**

Fragile X Syndrome (FXS) occurs as a result of a silenced fragile X mental retardation 1 gene (*FMR1*) and subsequent loss of fragile X mental retardation protein (FMRP) expression. Loss of FMRP alters excitatory/inhibitory signaling balance, leading to increased neuronal hyperexcitability and altered behavior. Acamprosate (the calcium salt of N-acetylhomotaurinate), a drug FDA-approved for relapse prevention in the treatment of alcohol dependence in adults, is a novel agent with multiple mechanisms that may be beneficial for people with FXS. There are questions regarding the neuroactive effects of acamprosate and the significance of the molecule’s calcium moiety. Therefore, the electrophysiological, cellular, molecular, and behavioral effects of acamprosate were assessed in the *Fmr1* ^*-/y*^ (knock out; KO) mouse model of FXS controlling for the calcium salt in several experiments.

**Methods:**

*Fmr1 KO* mice and their wild-type (WT) littermates were utilized to assess acamprosate treatment on cortical UP state parameters, dendritic spine density, and seizure susceptibility. Brain extracellular-signal regulated kinase 1/2 (ERK1/2) activation was used to investigate this signaling molecule as a potential biomarker for treatment response. Additional adult mice were used to assess chronic acamprosate treatment and any potential effects of the calcium moiety using CaCl_2_ treatment on behavior and nuclear ERK1/2 activation.

**Results:**

Acamprosate attenuated prolonged cortical UP state duration, decreased elevated ERK1/2 activation in brain tissue, and reduced nuclear ERK1/2 activation in the dentate gyrus in KO mice. Acamprosate treatment modified behavior in anxiety and locomotor tests in *Fmr1* KO mice in which control-treated KO mice were shown to deviate from control-treated WT mice. Mice treated with CaCl_2_ were not different from saline-treated mice in the adult behavior battery or nuclear ERK1/2 activation.

**Conclusions:**

These data indicate that acamprosate, and not calcium, improves function reminiscent of reduced anxiety-like behavior and hyperactivity in *Fmr1* KO mice and that acamprosate attenuates select electrophysiological and molecular dysregulation that may play a role in the pathophysiology of FXS. Differences between control-treated KO and WT mice were not evident in a recognition memory test or in examination of acoustic startle response/prepulse inhibition which impeded conclusions from being made about the treatment effects of acamprosate in these instances.

**Electronic supplementary material:**

The online version of this article (doi:10.1186/s11689-017-9184-y) contains supplementary material, which is available to authorized users.

## Background

Fragile X syndrome (FXS) is typically the result of a hypermethlyated cytosine-guanine-guanine (CGG) trinucleotide repeat expansion in the 5’ UTR of the Fragile X mental retardation 1 gene (*FMR1*), leading to its silencing and subsequent loss of its protein product, fragile X mental retardation protein (FMRP). FXS is the most prevalent, known single gene cause of developmental disability and autism spectrum disorder (ASD), occurring in 1:4000 males and 1:4000–6,000 females [1, 2]. FXS has a broad range of interfering phenotypic features including attention-deficit/hyperactivity disorder (ADHD) symptoms, aggression, self-injurious behavior, obsessive compulsive disorder-like behavior, hyperarousal to sensory stimuli, perseverative language, sleep issues, increased anxiety, increased risk for seizures, social and communication difficulties, and impaired cognition [3–5]. It is believed that these symptoms can largely be attributed to an altered balance in excitatory and inhibitory (E/I) neurotransmission in the FXS brain due to FMRP’s roles in synaptic plasticity and activity-dependent protein translation.

The E/I imbalance associated with FXS is driven, in part, by an increase in glutamatergic signaling events through group I metabotropic glutamate receptors (mGluRs), specifically mGluR5 [6–10]. Along with increased excitatory signaling, FXS is also characterized by reductions in γ-aminobutyric acid (GABA) signaling. Deficits in GABAergic signaling including reduced expression of GABA(A) receptor subunits, changes in the expression of the GABA synthesizing enzymes, and impaired tonic and phasic inhibition have been found in various brain regions including hippocampus, striatum, amygdala, and cortex in the *Fmr1* ^*-/y*^ (knock out; KO) mouse model of FXS [11–15]. FXS-associated alterations in the density and maturity of dendritic spines may also contribute to the E/I imbalance since these cellular components contain the post-synaptic elements of most glutamatergic synapses. Early reports in post-mortem, FXS human, Golgi-Cox stained tissue demonstrated an increased spine density and an abundance of immature appearing spines [16–18]. These results were also observed in subsequent studies of Golgi-Cox stained tissue from *Fmr1* KO mice [9, 19–23]. *Fmr1* KO mice also exhibit an increased duration of persistent cortical activity, or UP states, and decreased synchrony of inhibitory activity in response to thalamic stimulation, in line with elevated excitation and reduced inhibition [24]. It has also been shown that the increased UP state duration can be reversed through the genetic reduction of mGluR5 expression in *Fmr1* KO mice [25]. Juvenile *Fmr1* KO mice are also more susceptible than wild-type (WT) mice to audiogenic seizures, further supporting dysregulation in E/I balance in these mice [26].

Increased glutamatergic signaling and glutamate binding at mGluRs, which is observed in FXS, can modulate synaptic plasticity and gene transcription through activation of the extracellular signal-regulated kinase 1 and 2 (ERK1/2) pathway and lead to altered behavior [27]. ERK1/2 are central elements of intracellular signaling governing neuronal development [28, 29], synaptic plasticity [30], and memory formation [31], which are all processes altered in FXS. The isoforms, ERK1, and ERK2, exhibit significant functional redundancy and are thought to have resulted from single gene duplication at the onset of vertebrate evolution [32]. Both exhibit a similar three dimensional structure and are ubiquitously expressed in mammals with similar specific activity [33, 34]. ERK1/2 are activated by phosphorylation at threonine and tyrosine residues within their activation loop by upstream mitogen-activate protein kinase kinases, MEK1, and MEK2, leading to ERK1/2-facilitated transduction of extracellular signals [35]. ERK1/2 activation has been shown to be elevated in *Fmr1* KO mouse brain tissue, mouse blood lymphocytes, and can be attenuated by treatment with mGluR5 antagonists in mice [7, 36, 37]. Furthermore, brain ERK1/2 activation levels have been shown to be elevated in humans with FXS (post-mortem), and human blood lymphocyte activation kinetics are responsive to lithium therapy, suggesting that ERK1/2 alterations in FXS may be amenable to pharmacological treatment [38, 39]. Open-label acamprosate treatment in persons with FXS has been shown to modulate amyloid precursor protein (APP) and brain-derived neurotrophic factor (BDNF), both upstream regulators of ERK1/2 signaling [40–44]. ERK1/2 activation has been implicated in various seizure models and is also thought to play a role in *Fmr1* KO mouse audiogenic seizure susceptibility, further linking this signaling pathway with E/I imbalance and suggesting a central role in the pathophysiology of FXS [39, 45, 46].

Over the past 10 years, significant effort in FXS treatment development has focused on attenuating this E/I imbalance in the FXS brain. Recently in FXS clinical study, novel drugs specifically targeting a single receptor system involved in maintaining E/I balance, namely mGluR5, α-amino-3-hydroxy-5-methyl-4-isoxazolepropionic acid receptor (AMPA), or GABA(B) receptors, have been unsuccessful in clinical trial development [47]. Large-scale placebo-controlled trials have not demonstrated robust clinical improvement at the chosen doses, in the ages tested, and with the primary outcome measures utilized [48–50]. Acamprosate, an FDA-approved drug for the maintenance of alcohol abstinence, has pleotropic effects at multiple receptors and molecular signaling cascades that are disrupted in FXS, and has a good safety profile. Data within the alcoholism literature suggest that this drug could attenuate or reverse multiple points of glutamatergic dysfunction, potentially leading to improved E/I balance and ultimately improved behavior in FXS individuals [51, 52]. Although the exact mechanisms of acamprosate are unknown, and despite claims that the activity of acamprosate is due to calcium rather than N-acetylhomotaurinate [53], it is suspected to have pleotropic effects via mGlur5, GABA, and NMDA receptors to reduce neuronal hyperexcitability. Acamprosate has been demonstrated to bind at a spermidine-sensitive site at the N-methyl-D-aspartate (NMDA) glutamate receptor, have properties consistent with mGluR5 antagonism and GABA(A) agonism, and modulate dopamine release via glycine and nicotinic acetylcholine receptors [54–59].

Acamprosate has been assessed in several small open-label trials in FXS with benefits in the Clinical Global Impressions–Improvement (CGI–I) scale, as well as in other scales and checklists indicating improvements in social behavior and reductions in inattention/hyperactivity [41, 60]. Acamprosate is currently being investigated in a placebo-controlled trial in FXS (clinicaltrials.gov, NCT01911455). The current mouse studies were undertaken to identify electrophysiological, cellular, molecular, and functional changes associated with acamprosate treatment in the context of FXS and the E/I imbalance in the *Fmr1* KO mouse. Uncertainty regarding the calcium moiety of the acamprosate molecule and its effects on the drug’s neuroactivity is a critical question for future acamprosate drug development in FXS, and has come under debate in the chronic alcohol exposure field [53, 61–63]. Therefore, the contribution of the calcium moiety using CaCl_2_ treatment, controlling for the same number of Ca^2+^ ions as in the acamprosate dose, was also investigated in *Fmr1* KO and WT mice to determine the presence of any potential contribution to behavioral outcomes and ERK activation following chronic treatment.

## Methods

For the following experiments, two age groups were assessed: juvenile (P17-25; audiogenic seizure test and UP states) and adult (5–7 months; adult behavior and dendritic spine/ERK analyses). *Fmr1* KO mice (C57BL/6J background) are only susceptible to audiogenic seizures during early developmental periods. Additionally, UP state recordings are technically challenging when assessing adult brains and therefore we were unable to perform both of these tests at adult ages. Many of the behaviors assessed in the adult behavior battery are difficult to test during juvenile periods and therefore mice of adult ages were utilized for the behavior battery. Since acamprosate was ineffective at modulating juvenile behavior (seizures), but modulated adult behavior (elevated zero and locomotor behavior), efforts were concentrated on adult brain analyses (dendritic spine analyses, ERK activation).

## Neocortical slice preparation and UP state recordings

Spontaneous UP states were recorded from layer IV of acute neocortical slices prepared from male WT and *Fmr1* KO mice (P18-P25) on a C57BL/6J background as described previously [25, 64]. We [25] and others [65] have shown that UP state activity in layers IV and V is highly correlated. This is because UP states reflect the synchronous activity of populations of neurons and circuits in the cortex, so the layer IV and V neurons are firing relatively synchronously. In *Fmr1* KO slices, UP state duration is longer in both layers IV and V and are also highly correlated. We chose to measure layer IV UP states in this study because spontaneous, brief or non-UP state activity is greater in layer V and this contributes to a higher baseline “noise” which makes detection of UP state activity more difficult in layer V. In layer IV recordings, there is less inter-UP state activity and thus UP states are more accurately detected and measured. In the current experiment, 4 WT mice and 10 *Fmr1* KO mice were anesthetized with ketamine (125 mg/kg)/xylazine (25 mg/kg) and decapitated. The brain was transferred into ice-cold dissection buffer containing the following (in mM): 87 NaCl, 3 KCl, 1.25 NaH_2_PO_4_, 26 NaHCO_3_, 7 MgCl_2_, 0.5 CaCl_2_, 20 D-glucose, 75 sucrose, 1.3 ascorbic acid, and 1.5 Kinurenic acid aerating with 95% O_2_–5% CO_2_. Thalamocortical slices (400 μm) were made on an angled block [66] using a vibratome (Leica VT 1200 Plus). Thalamocortical slices were immediately transferred to an interface recording chamber (Harvard Instruments) and allowed to recover for 1 h in ACSF at 32 °C containing the following (in mM): 126 NaCl, 3 KCl, 1.25 NaH_2_PO_4_, 26 NaHCO_3_, 2 MgCl_2_, 2 CaCl_2_, and 25 D-glucose. The original observation of these maintained states was used with thalamocortical slices and using thalamically evoked UP states [24]. Even though thalamic connections to cortex are not required to observe UP states or to observe prolonged UP states in *Fmr1* KO mice, as determined in Hays et al. 2011, this is a common slice preparation.

For UP state recordings, 60 min before the beginning of a recording session, slices in the interface chamber were perfused with an ACSF that mimics physiological ionic concentrations in vivo [24, 65] and contained the following for vehicle (VEH)-treated slices (in mM): 126 NaCl, 5 KCl, 1.25 NaH_2_PO_4_, 26 NaHCO_3_, 1 MgCl_2_, 1 CaCl_2_, and 25 D-glucose. For the acamprosate-treated slices, the previous buffer was used to dilute acamprosate (N-acetylhomotaurinate; 3-(Acetylamino)-1-propanesulfonic acid hemicalcium salt; IND Swift Laboratories; USP) to a 200 μM concentration. Following the 60-min incubation with VEH or acamprosate buffer, spontaneously generated UP states were recorded using 0.5 MΩ tungsten microelectrodes (FHC) placed in layer IV of the somatosensory cortex (WT + VEH, *n* = 16; WT + Acamp, *n* = 14; KO + VEH, *n* = 27; WT + Acamp, *n* = 25 slices). 5 min of spontaneous activity was collected from each slice. Recordings were amplified 10,000× and filtered online between 500 and 3 kHz. All measurements were analyzed off-line using custom Labview software. For visualization and analysis of UP states, traces were offset to zero, rectified, and low-pass filtered with a 0.2 Hz cutoff frequency. The threshold for detection was set at 5× the root mean square noise. An event was defined as an UP state when its amplitude remained above the threshold for at least 200 ms. The end of the UP state was determined when the amplitude decreased below threshold for >600 ms. Two events occurring within 600 ms of one another were grouped as a single UP state. UP state amplitude was defined based on the filtered/rectified traces and was unit-less because it was normalized to the detection threshold. This amplitude may be considered a coarse indicator of the underlying firing rates of neuronal populations. UP state duration, amplitude, and number of events were analyzed by two-way ANOVA with gene (KO, WT) and drug (VEH, 200-μM acamprosate (+Acamp)) as factors. Pairwise comparisons were performed and corrected with FDR (two-tailed).

## Mice for in vivo treatment studies

For dendritic spine quantification, ERK1/2 activation, and behavior studies, a breeding colony of *Fmr1* KO mice [67] was established in the Rodent Barrier Facility at Cincinnati Children’s Research Foundation (CCRF). All protocols were approved by the CCRF Institutional Animal Care and Use Committee. Animals were maintained with regulated light cycles (14:10 h light:dark cycle, lights on at 600 h) with controlled temperature (19 ± 1 °C) and humidity (50 ± 10%). Test subjects were generated from the mating of female *Fmr1* ^*+/−*^ mice to male WT mice on a C57BL/6J background. Mice from these pairings were used as test subjects for all experiments except UP state recordings (described above). Mice were genotyped on postnatal day (P) 10 by ear clip and weaned on P28. Adult male *Fmr1* KO and WT littermates were used for experiments and group housed throughout testing (2–4 per cage).

## Juvenile audiogenic seizure test

Male *Fmr1* KO and WT littermates were housed with their litter and dam, and were treated via intraperitoneal (IP) injection with saline (SAL; USP) or 500 mg/kg acamprosate (expressed as the free base) once per day (10 ml/kg dosing volume) from P17*–*21 (*n* = 13–17 per group). 30 min following the fifth dose on P21, mice were assessed in an audiogenic seizure test which consisted of a two-minute priming tone (120-dB siren), which does not typically induce seizure behavior, followed by 1 min of silence and then a second tone (120-dB siren) lasting an additional 2 min. Each mouse was tested alone in a static mouse cage free of bedding. A Mugger Stopper Plus personal alarm was used to generate the tone and was placed on the filter cage lid with the speaker facing down into the cage. The battery was replaced often to ensure the sound intensity was always at maximum. During the second tone, behavior response was scored as 0, 1, 2, 3, or 4 describing the least severe response of 0 indicating no altered behavior, followed by 1 indicating wild-running, 2 indicating clonic seizure (rapid limb flexion and extension), 3 indicating tonic seizure (static limb extension), and 4 indicating the most severe response of cardiac arrest [68]. No seizure behavior was observed during the priming tone for this cohort of mice. Seizure severity during the second tone was calculated by using an animal’s most severe response number. Seizure severity was analyzed by the Exact Wilcoxon Rank sum test for non-parametric data. Treatment group (WT + VEH, WT + Acamp, KO + VEH, and KO + Acamp) was used with exact probabilities calculated to determine pairwise group comparisons. These group comparisons were corrected using the FDR method.

## Dendritic spine and ERK1/2 quantification

Male *Fmr1* KO and WT littermates (6–7 months old) received once daily treatment (10 ml/kg volume) with 300 mg/kg acamprosate (expressed as the free base; IND-Swift Laboratories; USP) or USP saline vehicle (SAL) for 26 days and were sacrificed 1 h following their last dose (6 mice per group). These mice were used to pilot behavior studies in *Fmr1* KO mice with acamprosate treatment, but were not included in the adult behavior analysis due to modified behavior protocols used in the adult behavior battery described below and the small number of mice tested in this group. Mice for ERK1/2 and spine analyses were not handled for 3–5 days prior to sacrifice with the exception of the continued once daily IP treatment injection. Particular care was taken to minimize stress on the final day of treatment and mice were removed from their cage, which was kept in their permanent housing room and transferred directly to necropsy one at a time. Decapitation occurred within 30 sec from removal of the mice from the housing room. Brains were removed and maintained on ice. For ERK1/2 determinations, the hippocampus and a 1-mm-thick section of striatum were removed from one hemisphere and rapidly frozen onto a stainless steel plate over dry ice. Once frozen, brain tissue was transferred to a microfuge tube and stored at −80 °C until assayed. The remaining hemisphere was rinsed with Milli-Q water and immersed in the impregnation solution to begin the Golgi staining process (see below).

### Dendritic spine quantification

One hemisphere per animal (5 animals per treatment group) was processed for Golgi staining using FD Rapid GolgiStain™ Kit (FD NeuroTechnologies Inc.) according to manufacturer instructions. Golgi-Cox stained brains were sectioned at 150 μm thickness onto gelatin-coated slides using a cryostat, processed according to manufacturer’s directions, and coverslipped in DPX mounting medium. Five layer V pyramidal neurons from the somatosensory cortex with intact apical dendrites extending at least 150 μm from the soma were selected from each animal (*n* = 25 cells per treatment group). Due to the nature of staining and method of cell counting, cells with isolated dendrites (not overlapping with other cell processes) were preferentially chosen so that overlapping areas did not impede spine counting. Z stacks containing the apical dendrite were obtained using an upright bright-field microscope (Zeiss Axioplan 2; Axiovision software 4.8) equipped with a 40× oil immersion objective, with a Z step of 0.15 μm, which typically generated 250 optical sections for each cell. Each apical dendrite was subdivided into six 25-μm-long segments, and dendritic spines were counted manually using Neurolucida (MBF Bioscience) tracing software while scrolling through the Z stacks. Data were analyzed by three-way mixed factor ANOVA with gene and drug as between factors and segment as a within factor. Slice effects and pairwise comparisons with FDR adjustment were performed.

### ELISA quantification of ERK1/2 activation

For total protein determination, the hippocampus and striatum were homogenized in ice-cold RIPA buffer (500 and 100 μl, respectively) with the fresh addition of HALT phosphatase inhibitor cocktail (ThermoScientific) and protease inhibitor cocktail (Sigma) and assayed using the Pierce BCA Protein Assay Kit (ThermoScientific) according to manufacturer’s instructions. Samples were diluted to 50 μg/ml for phosphorylated ERK1/2 (pERK1/2) and 2.5 μg/ml for ERK1/2 total prior to analysis. pERK1/2 and ERK1/2 total were analyzed by semiquantitative SimpleStep ELISAs (enzyme-linked immunosorbent assay; ABCAM; phosphoERK1/2 pT202/Y204, ab176640 and ERK1/2 total, ab176641) according to manufacturer’s instructions. Briefly, supplied concentrated capture and detector antibody was diluted in supplied antibody dilution buffer. Standards were prepared as directed and 50 μl of samples and standards were added to each well and assayed in duplicate. The optical density (OD) was read at 450 nm. Data were verified to fall within the linear range of the standard curve. These ELISAs are semiquantitative with standards supplied at an unknown concentration of phosphorylated recombinant ERK protein and do not allow for the exact concentration of pERK1/2 or ERK1/2 total. Therefore, mean OD of duplicate samples was used for calculations. ERK1/2 total and the ratio of pERK1/2 over ERK1/2 total normalized to WT + SAL were analyzed by two-way ANOVA with genotype (WT or Fmr1 KO) and drug (SAL, 300 mg/kg acamprosate) as factors. For pERK/ERK total, a priori comparisons between the WT + SAL and KO + SAL groups, and the KO + SAL and KO + Acamp groups were performed with predictions of increased pERK/ERK total ratio in the KO + SAL group compared to the WT + SAL control, and decreased ratio in the treated KO mice compared to SAL-treated KO group in both the striatum and hippocampus. All pair-wise comparisons were corrected using FDR.

### pERK/NeuN immunostaining

60 min following a final treatment dose (2 days following the completion of the adult behavior battery), the animals were deeply anesthetized with pentobarbital and transcardially perfused with 5-mL ice-cold 1× PBS followed by 4% PFA. Whole brains were sectioned coronally using a Leica SM2000R freezing, sliding microtome at 35 μm. Tissue sections were bleached in 3% H_2_O_2_ for 30 min. Sections were then blocked in 10% normal donkey serum (NDS) for 1 h. Sections were incubated in 1:400 rabbit, anti-pERK1/2 primary antibody (#4370; Cell Signaling) for 48 h followed by incubation in 1:200 swine, anti-rabbit, biotinylated secondary antibody (E0353; Dako) solution for 3 h. Following secondary, tissue was incubated for 1 h in ABC solution (VECTASTAIN Elite ABC HRP Kit; Vector) which was prepared 30 min prior to use. Tissue was then incubated in tyramide biotin solution prepared in 0.1-M Borate buffer, pH 8.0 with 0.003% H_2_O_2_ for 10 min. Tissue was then incubated with 1:200 Alexa 488 conjugated streptavidin (Jackson ImmunoResearch) for 2 h. Sections were then placed in 1:500 mouse, anti-NeuN primary antibody (MAB377; Milllipore) solution overnight. Sections were then incubated in 1:200 donkey anti-mouse Alexa 594 conjugated secondary antibody (Jackson ImmunoResearch) for 2 h. All steps were performed at room temperature. Sections were washed between incubations 3 times in 1× KPBS with 0.2% Triton X-100 for 10 min per wash. All antibody solutions were prepared in 1× KPBS with 0.2% Triton X-100 and 2% NDS. Images were acquired using a Nikon A1 inverted, single photon, confocal microscope, using a 4× objective with pixel size minimized to the Niquist limit. Images were taken from sections at −2.5 mm from Bregma, and pERK1/2 positive cells were identified using the General Analysis functionality in NIS-Elements. ROIs were then manually applied and pERK1/2 positive nuclei were automatically counted using NIS-Elements. Neuronal identity of cells was assessed by colocalization of pERK1/2 with NeuN.

## Adult behavior battery

### Drug treatment

For the groups of mice that were assessed in the adult behavior battery (and subsequent pERK1/2 immunostaining), male WT and *Fmr1* KO littermates (5–7 months old) were randomly assigned to a treatment group and treated once daily with 0 (SAL vehicle), 300 mg/kg of acamprosate calcium (expressed as the free base), or 122.2 mg/kg calcium chloride USP (CaCl_2_ × 2H_2_O; Sigma-Aldrich) in a volume of 10 ml/kg via IP injection. Note that calcium salt and acamprosate calcium contained equivalent amounts of Ca^2+^ ions (0.8 mmol/kg/day). Dosing commenced 10 days prior to, and continued throughout behavior testing. Drug treatment occurred between 0900 and 1100 h with an interval of 60 min between drug treatment and the start of behavior assessment each day. Mice were treated for a total of 21 days (9–13 mice per treatment group were tested). Adult behavior analysis was completed in two separate cohorts with genotype and drug group combinations balanced across cohorts. Data are shown as single treatment groups since no differences between cohorts were apparent.

### Dose selection

The dose used in the current study was based on previously published reports in rodents which demonstrated that > 100 mg/kg was needed to reduce alcohol craving and nicotine-seeking behavior, and 200 mg/kg was required to improve transient hemispheric ischemia-induced neurological deficits [69, 70]. The therapeutic dose of acamprosate for alcohol withdrawal and the current adult FXS treatment dose is ~2 g/day for an average 70 kg human subject (equivalent to 28.5 mg/kg). Using the human equivalent dose based on body surface area calculation for inter-species dose scaling, the daily mouse adult behavior battery dose (300 mg/kg; free base) is equivalent to 1.9 g/day in a 70 kg human ((333 mg/kg × 3/37 (mouse to human ratio) = 27) × 70 kg adult = 1.9 g dose).

### Behavior analysis

Behavior was assessed during the light portion of the light/dark cycle, and food and water were available ad libitum except during behavior testing. Mice began testing on day 11 of treatment. To minimize the impact of stress during behavioral testing, mice were transported across the hallway to the Rodent Behavior Core and dosed with SAL, CaCl_2_, or acamprosate and allowed at least 60 min in the testing room to acclimate before behavior assessment daily. Elevated zero maze was the only exception in which mice were brought into the testing room one at a time just prior to being placed on the maze in order to get an accurate anxiety assessment. Animals were tested in only one paradigm per day and were given at least 1 day of rest in between each test (drug treatment continued even on resting days). Behavior was evaluated in the following order so that tests easily influenced by stress were completed early during the behavior battery: elevated zero maze, locomotor activity, novel object recognition, acoustic startle habituation, and prepulse inhibition. Apparatus surfaces were cleaned with Process NPD (Steris) before and between animals.

### Elevated zero maze (EZM)

The EZM was used to assess anxiety-like behavior as previously described with modification of the maze size [71]. Briefly, mice were transported from the housing room to the testing room individually and placed on the apparatus. The experimenter exited the room immediately after placing the mouse in one of the closed quadrants of the apparatus. A camera mounted above the maze connected to a computer located outside the room was used to observed and score, in real-time, time in open quadrants, number of head dips, number of open arm entries, and latency to first enter an open quadrant during a single 5 min trial (ODLog, Macropod Software). The test room was dimly lit (30 lux (lx)) to encourage exploration of the test environment. Two mice were removed from the EZM analysis after falling from the maze.

### Locomotor activity

Activity analysis in an open field, an overall indication of an animal’s activity level, is sensitive to sedative drugs or those inducing stereotypy or catatonia, and is especially useful in better interpreting other tasks that depend on the overall activity of the animal. Locomotor activity was measured in infrared photocell activity chambers (41 × 41 cm; PAS Open Field, San Diego Instruments, San Diego, CA) for 1 h. Number of beam breaks was recorded during 5 min intervals for a total of 12 intervals and analyzed by three-way ANOVA with repeated measures. Room lights were at full level (1200 lx).

### Novel object recognition (NOR)

A solid black enclosure with dimensions 19.5 cm L×40 cm W×35 cm H was used to assess NOR. During the familiarization phase, mice were presented with two identical objects for a total of 5 min. Mice were returned to their cage and left undisturbed for 30 min. Next, mice were placed back in the enclosure with a novel object and one identical copy of the familiarization phase objects. Pilot mice were previously shown to have no inherent preference for familiar or novel objects used in this test (data not shown). The amount of time each mouse spent paying attention to the familiar and novel objects during the familiarization and test phases was recorded using OD Log (Macropod Software) for the 5 min duration of each phase. Time spent paying attention was recorded when the mouse was oriented toward the object with snout within 1 cm of the object or when forepaws were up against the object. Mice in these cohorts did not climb on top of the objects used for this test. The discrimination index (DI; novel object time—familiar object time/novel object time + familiar object time) was used to determine the degree of object memory. Dim lighting conditions (20 lx) were used to reduce anxiety and encourage object exploration during both phases. Six mice were removed from the NOR analyses due to accumulating less than 6 s of total time paying attention to the objects during the test phase. Total exploration time and DI during the test phase were analyzed separately by two-way ANOVA.

### Acoustic startle habituation and prepulse inhibition (PPI)

Acoustic startle habituation and PPI were assessed in a sound-attenuating test chamber (SR-LAB apparatus; San Diego Instruments, San Diego, CA) as previously described with modifications [72]. Mice were placed in an acrylic cylindrical holder that was mounted on a platform with a piezoelectric force transducer attached to the underside of the platform. For both habituation and PPI, a 5 min acclimation period preceded test trials. For habituation, each animal received 50 repeated 20 ms 120 dB SPL mixed frequency sound bursts (1.5 ms rise time). Maximum velocity for each trial (V_max_; measured in arbitrary units; a.u.) was analyzed by repeated measures three-way ANOVA. For PPI, each animal received a 5×5 Latin square sequence of trials that were of five types: startle stimulus (SS) with no prepulse (PPI0), no SS with no prepulse, 73 dB prepulse + SS, 77 dB prepulse + SS, or 82 dB prepulse + SS. The startle signal was a 20 ms 120 dB SPL mixed frequency sound burst (1.5 ms rise time). Prepulses preceded the startle-eliciting stimulus by 70 ms (onset to onset). The startle recording window was 100 ms. Background noise level was 70 dB. Each set of 25 trials was repeated 4 times for a total of 100 trials. The inter-trial interval averaged 14 s and varied randomly from 8–20 s. Percent PPI was calculated as (100*(V_max_ at PPIxx/max velocity PPI0) for the PPI trials. Percent PPI at each prepulse level was analyzed by three-way mixed factor ANOVA with gene and drug as between factors and PPI Trial Type as a within factor (Table 1). Two mice were removed from the startle habituation analysis and one removed from the PPI analysis due to equipment errors in data recording (i.e., no data recorded by software).

**Table 1:** Summary of baseline control-treated KO and WT effects and KO acamprosate treatment effects

Baseline effects KO_Controls vs. WT_Controls	KO acamprosate treatment effects KO + Acamp vs. KO_Controls
Increased UP state duration^*^	Decreased UP state duration^*^
Increased seizure severity score^*^	No treatment effect on seizure severity score
Increased pERK/ERK total ratio HIP^*^, STR^*^ lysate	Decreased pERK/ERK total ratio HIP^*^, STR^*^ lysate
Increased pERK1/2+ cell counts^†^	Decreased pERK1/2+ cell counts^*^
Increased EZM time in open^*^	Increased EZM time in open^*^
Increased locomotor activity^*^	Decreased locomotor activity^*^

*ERK1/2*, extracellular signal-related kinase 1/2; *pERK1/2*, phosphorylated ERK1/2; *EZM*, elevated zero maze; *STR*, striatum; *HIP*, hippocampus. ^*^ *p* < 0.05, ^†^ *p* < 0.1.

## Statistics

All data were analyzed using mixed linear factorial analysis of variance (ANOVA; Proc Mixed) with the exception of seizure severity score in which the Exact Wilcoxon Rank sum for non-parametric data was used (SAS v9.2, SAS Institute, Cary, NC). Significant main effects and interactions were followed-up with pairwise group comparisons using the false discovery rate (FDR) method to control for multiple comparisons [73]. Specific details relating to between and within factors, preplanned tests, and repeated measures were briefly described above with specifics detailed in the Results. All behavioral coding, slice analyses, spine counting, and molecular assays were performed by experimenters blind to genotype and treatment group. Data are shown as least squares (LS) mean ± standard error of the mean (SEM) for model consistency with the exception of seizure severity, in which ordinary means and SEM are shown. A *p* value of less than 0.05 was considered significant and trends are reported at *p* < 0.1.

For the adult behavior battery and subsequent pERK1/2 immunostaining, an initial analysis was performed for each measure to determine if there were differences between the SAL- and CaCl_2_-treated control groups (F ratios listed in table format (Additional file 1: Tables S1 (two-way ANOVAs) and S2 (three-way ANOVAs)). No differences in any behavior or immunostaining measure were detected with SAL and CaCl_2_ treatment (Additional file 1: Figure S1) and therefore these groups were combined for the final analyses with significant and trending main effects and interaction statistics shown in the text with ‘control combined’ F ratios listed in table format (Additional file 1: Tables S3 (two-way ANOVAs) and S4 (three-way ANOVAs)).

## Results

### UP state recording

**Fig. 1:**
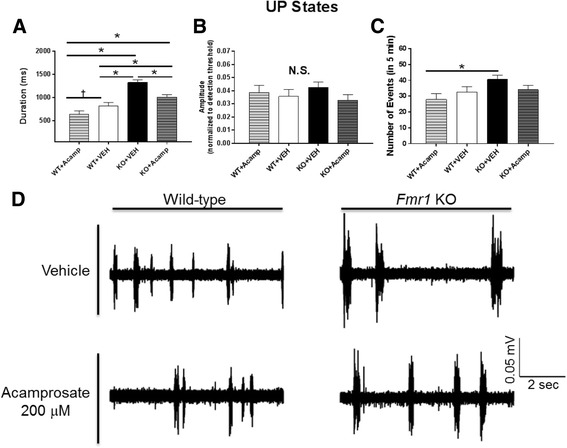
UP state recordings. Spontaneous UP states were measured in slices from P18–25 mice for 5 min in layer IV of the somatosensory cortex. Duration (**a**), amplitude (**b**), and number of events (**c**) were analyzed by two-way ANOVA with pairwise comparisons corrected using FDR method (two-tailed). Representative traces are shown in panel (**d**). There was a significant increase in UP state duration in the KO + VEH-treated mice compared to the WT + VEH-treated mice indicating a baseline effect of genotype. Bath application of 200 μM acamprosate significantly decreased the elevated UP state duration in the KO mice indicating a significant treatment although the acamprosate-treated KO slices still had UP state durations that were longer than WT + VEH slices. There was a trend towards a decreased UP state duration in the WT + Acamp group compared to the WT + VEH group. For number of events, there was a main effect of gene, and the KO + VEH slices had more UP state events than the WT + Acamp-treated mice. No change in amplitude was observed. WT + VEH, *n* = 16; WT + Acamp, *n* = 14; KO + VEH, *n* = 27; KO + Acamp, *n* = 25 slices; data shown are LS mean ± SEM; *p < 0.05, †*p* < 0.1; *N.S.* = not significant

Juvenile *Fmr1* KO neocortical circuits are hyperexcitable as revealed by the long duration of spontaneous persistent, activity, or UP states of neuron networks [25]. Here we measured UP states with extracellular, multiunit recordings in layer IV of acute slices of somatosensory, or barrel, neocortex from WT or *Fmr1* KO mice littermates with bath application of acamprosate or vehicle (Fig. 1a). Duration and amplitude for each UP state as well as the number of UP states during the five-minute time-period were analyzed by two-way ANOVA (Additional file 1: Table S3) with pairwise differences corrected using FDR (two-tailed; Fig. 1). For duration of UP states (Fig. 1a), there was a significant main effect of gene (ANOVA, F(1, 78) = 4.71, *p* = 0.0001) and drug (ANOVA, F(1, 78) = 15.74, *p* = 0.0002). As previously reported [25], UP state duration was greater in the KO + VEH group compared to the WT + VEH group (*p* = 0.0002). Acamprosate treatment in the KO mice reduced this increase compared to the KO + VEH (*p* = 0.0002), although this was still slightly elevated compared to the WT + VEH mice (*p* = 0.049; see Fig. 1d for representative traces). Acamprosate treatment in the WT mice produced a trend towards a decrease in duration compared to the WT + VEH group (*p* = 0.071) and a significant decrease compared to the KO + VEH (*p* = 0.0002) and the KO + Acamp groups (*p* = 0.0002). No significant effects were found for amplitude normalized to detection threshold (Fig. 1b). For number of events in 5 min (Fig. 1c), there was a main effect of gene (ANOVA, F(1, 78) = 5.14, *p* = 0.026) although pairwise differences were not evident in pertinent group comparisons (WT + Acamp vs. KO + VEH group (*p* = 0.035)). These data indicate that hyperexcitability of neocortical circuits in the developing *Fmr1* KO mice, as measured by prolonged UP states, is improved by acamprosate treatment.

### Audiogenic seizure test

**Fig. 2:**
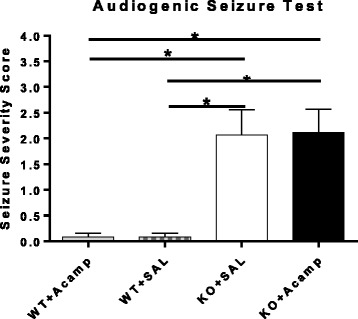
Audiogenic seizure test. Audiogenic seizure severity was assessed in juvenile WT and KO mice after 5 days of treatment. The test was performed 60min after mice received the final dose. Both KO groups had increased seizure severity scores compared to each WT group with no effect of acamprosate treatment on seizure severity in either genotype (Wilcoxon rank sum test with exact probabilities calculated to determine pairwise group comparisons; FDR corrected). WT + SAL (*n* = 13), WT + Acamp (*n* = 13), KO + SAL (*n* = 15), KO + Acamp (*n* = 17); data shown are mean ± SEM; *p < 0.05

Juvenile *Fmr1* KO mice are susceptible to audiogenic-induced seizures although WT mice (B6 background) of all ages and adult KO mice are resistant. A pilot experiment using 300 mg/kg acamprosate failed to attenuate seizure susceptibility (data not shown) and therefore the higher dose of 500 mg/kg was chosen for this experiment. In the current study, seizure severity score was analyzed in P21 *Fmr1* KO and WT littermates following 5 days of SAL or acamprosate (500 mg/kg) treatment using the Wilcoxon statistic, *S* = 175.5, and demonstrated a significant effect of treatment group (*p* = 0.0004) (Fig. 2). Exact probabilities were computed to determine pairwise comparisons corrected using FDR (two-tailed) and revealed significant increases in seizure severity score in both FXS groups compared to each WT group (*p* = 0.003 for each comparison). No within-genotype differences were detected indicating acamprosate treatment did not alter seizure severity in either the WT or KO mice, although a baseline difference was detected between control-treated KO and WT mice as expected.

### Dendritic spine quantification

**Fig. 3:**
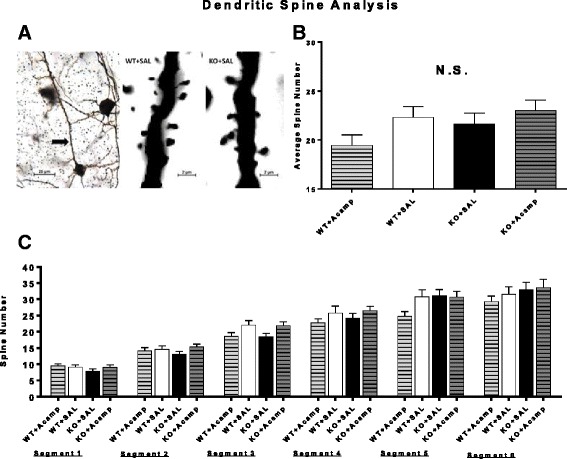
Dendritic spine density. Representative image of a layer V pyramidal neuron in the somatosensory cortex meeting the selection criteria for dendritic spine quantification (**a**, *left panel*; *arrow* indicating apical dendrite; scalebar = 25 μm) and representative cropped images from single focal planes demonstrating dendritic spine resolution power of microscopy technique (**a**, *middle panel*: WT + SAL; right panel: KO + SAL; scalebar = 2 μm). Apical dendritic spines were counted in layer V pyramidal neurons in the somatosensory cortex of 7-month-old male WT and KO mice following 26 days of treatment with SAL or acamprosate (300 mg/kg). Data were analyzed by a three-way mixed factor ANOVA with gene and drug as between factors and segment as a within factor. There was a significant main effect of segment and interactions of gene×drug (**b**) and drug×segment were approaching but did not reach significance. As expected, the number of spine counts increased in all groups as distance increased from the soma (**c**). Data shown are LS mean ± SEM; *p < 0.05; †*p* < 0.1

A three-way mixed factor ANOVA with gene and drug as between factors and segment as a within factor (Additional file 1: Table S4) was used to analyze spine number along the first 150 μm length of apical dendrites divided into six 25 μm segments from layer V pyramidal neurons located in the somatosensory cortex in adult mice (*n* = 25 cells/group). There was a significant main effect of segment (ANOVA, F(5, 460) = 87.36, *p* = 0.0001) in which the number of spines in all groups increased as a function of distance from the soma (Fig. 3c). Gene × drug (Fig. 3b) and drug x segment interactions were only approaching significance and therefore additional post hoc analyses were not completed. These data indicate that there were no observable spine differences detected between the control-treated KO and WT mice and therefore no deficit for acamprosate to modulate.

### ERK1/2 activation

**Fig. 4:**
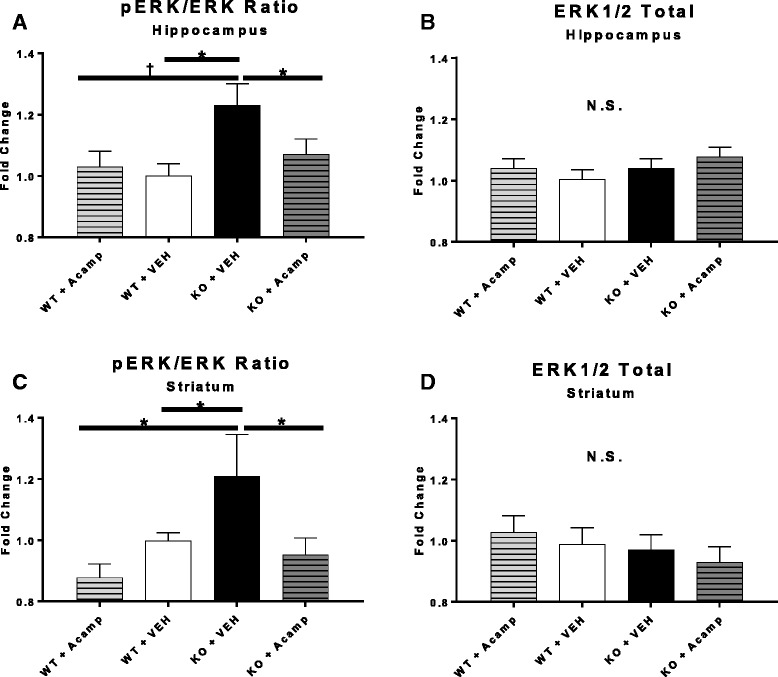
ERK1/2 activation ratios. In the hippocampus (**a**, **b**) and striatum (**c**, **d**) ERK1/2 activation ratios (pERK/ERK total) were calculated (*left panels*) as well as ERK1/2 total protein expression (*right panels*) with data normalized to the WT + SAL group. Data were analyzed by two-way ANOVA and pairwise comparisons corrected with FDR. A significant increase in pERK/ERK total ratio was found in the KO + SAL group compared to the WT + SAL group in the hippocampus and striatum (one-tailed) as predicted. The pERK/ERK total ratio increase in the KO + SAL group was also evident when compared to the WT + Acamp group (two-tailed). In both brain regions, chronic treatment with acamprosate (300 mg/kg) reduced pERK/ERK total ratios in the KO mice to a level not distinguishable from WT + SAL mice (one-tailed) as predicted. There were no differences in the amount of ERK1/2 total in either brain region or between any groups. *n* = 6 per group and brain region; data shown are LS mean ± SEM; *p < 0.05, †*p* < 0.1; *N.S.* = not significant

Separate two-way ANOVAs (Additional file 1: Table S3) were used to determine the effects of gene and drug and the interaction of gene × drug in the hippocampus and striatum on pERK/ERK total ratio and ERK1/2 total (each region was normalized to WT + VEH; *n* = 6 per group and brain region). All pairwise group comparisons were corrected using FDR. For ERK1/2 total absorbance, no significant main effects or interactions were identified in the hippocampus (Fig. 4b) or striatum (Fig. 4d), demonstrating that neither genotype nor drug altered ERK1/2 total protein expression. Therefore, group differences in ERK1/2 activation/phosphorylation are not influenced by baseline changes in total ERK1/2 expression and can be attributed to changes in ERK activation. For pERK/ERK total ratios, there was a significant main effect of gene in the hippocampus (ANOVA, F(1, 20) = 6.06, *p* = 0.023) (Fig. 4a) and a main effect of drug in the striatum (ANOVA, F(1, 20) = 5.89, *p* = 0.02) (Fig. 4c). We predicted baseline increases in pERK/ERK total ratios in the KO + SAL group compared to the WT + SAL group based on previous reports in which ERK1/2 activation has been shown to be elevated in the brains of *Fmr1* KO mice compared to WT mice [39, 74]. Furthermore, we predicted acamprosate treatment would decrease pERK/ERK total ratios based on data showing drugs with similar anti-glutamatergic actions to acamprosate significantly decreased aberrant ERK1/2 activation in *Fmr1* KO mice and decreased ERK1/2 activation kinetics in FXS patient blood samples [7, 75]. Because our a priori predictions were directional for these specific comparisons (WT + SAL vs. KO + SAL; KO + SAL vs. KO + Acamp), one-tailed tests were used for these specific ERK1/2 preplanned tests. Baseline comparisons showed a significant increase in pERK/ERK total ratio in the KO + SAL group compared to the WT + SAL group in both the hippocampus (*p* = 0.008) and striatum (*p* = 0.035) which is in line with previous reports. Preplanned comparisons between the KO + SAL and the KO + Acamp mice showed a reduction in pERK/ERK total ratio in both the hippocampus (*p* = 0.026) and striatum (*p* = 0.03) with acamprosate treatment as predicted. When comparing the KO + SAL-treated mice to the WT + Acamp-treated mice, there was a trend toward a pERK/ERK total increase in the hippocampus (*p* = 0.05) and a significant increase in the striatum (*p* = 0.04). No differences were noted in pERK/ERK total ratio in the hippocampus or striatum between the two WT groups (*p* = 0.71 and *p* = 0.43, respectively).

**Fig. 5:**
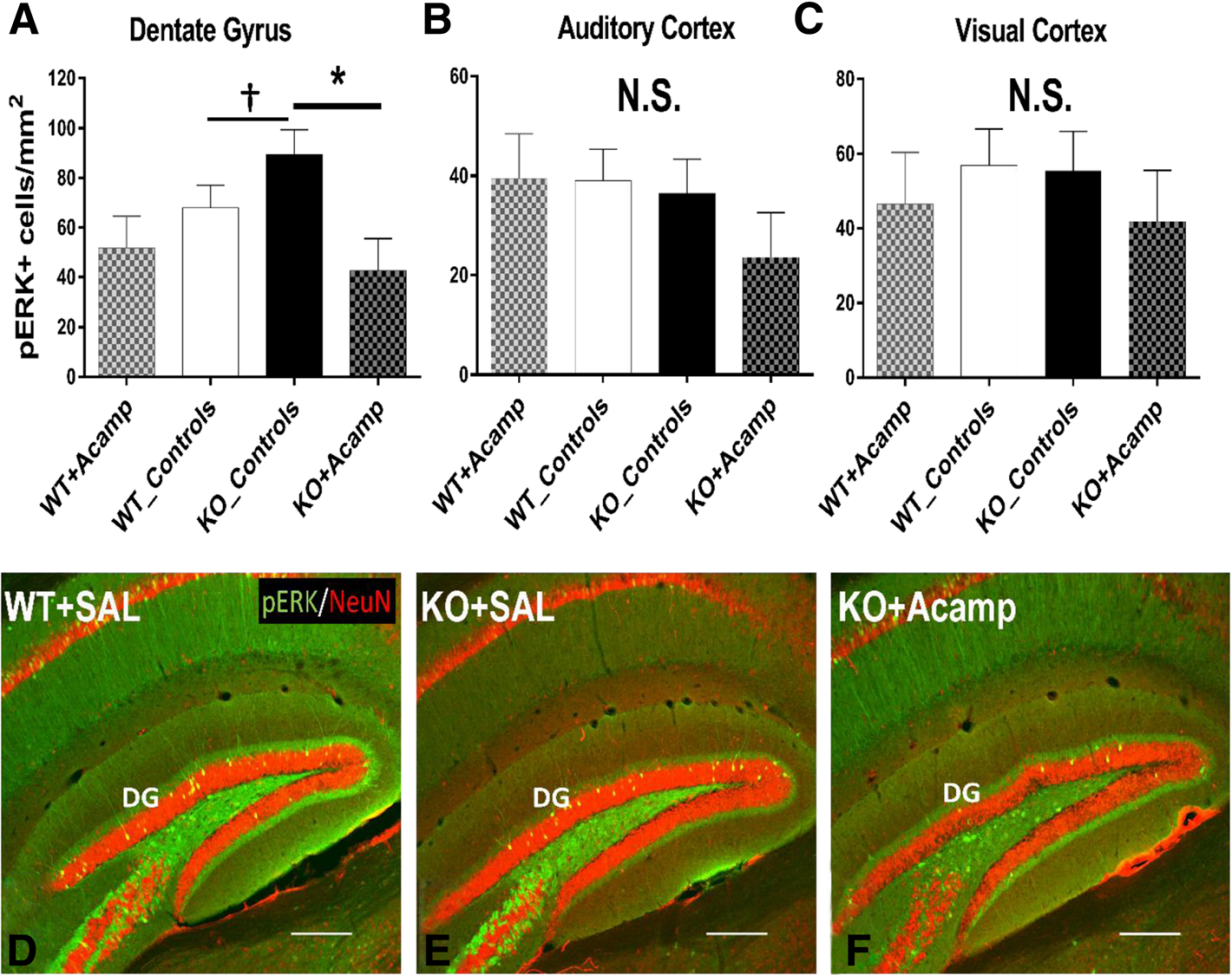
pERK1/2+ cell counts. Following the adult behavior battery (chronic treatment with saline (SAL) or 122.2 mg/kg CaCl_2_ in SAL (_Controls; equivalent amount of Ca^2^+ ions as in the 300 mg/kg acamprosate treated group) or 300 mg/kg acamprosate in saline (+Acamp)), mice were sacrificed and brain sections were stained for pERK1/2 (*green*) and NeuN (*red*). As with the behavior measures, there were no differences in pERK1/2+ cell counts between the SAL- and CaCl_2_-treated mice and therefore data are presented as combined control groups (controls). In the dentate gyrus (**a**, **d**–**f**), there was a significant effect of drug with pairwise comparison testing demonstrating a trend towards an increase in pERK1/2 positive cells in the KO_Controls group (KO + SAL pictured in **e**) compared to the untreated WT group (WT + SAL pictured in **d**). Additionally, the KO + Acamp group (**f**) had significantly fewer pERK1/2+ cells than the KO + Controls. In the DG, all pERK1/2+ cells were also NeuN+. There were no differences in PERK1/2+ cell counts observed in the auditory cortex (**b**) or in the visual cortex (**c**). Data shown are LS mean ± SEM; *p < 0.05; †*p* < 0.1; *N.S.* = not significant. *n* = 5–6 sections/group. Scalebar = 250 μm

To determine if acamprosate modulated ERK1/2 activity in a region/cell type specific manner, we immunostained brain sections from mice that completed the adult behavior battery. Data were first analyzed to determine if there were any within genotype differences in pERK1/2+ cell counts in mice treated with either SAL or CaCl_2_ and found no differences in the dentate gyrus (DG), auditory cortex, or visual cortex (Additional file 1: Figure S2). Since there were no effects of CaCl_2_ treatment on either WT or KO mice compared to SAL-treated mice, these groups were combined to create a single control group. A two-way ANOVA for cell counts revealed a main effect of drug (ANOVA, F(1, 30) = 7.59, *p* = 0.01) in the DG (Fig. 5a, e, f), but no effects in cortical regions (Fig. 5b, c). In the DG, baseline differences between genotypes in pERK1/2+ cell counts demonstrated a trend showing an increase in pERK1/2+ nuclei in the KO_Controls compared to the WT_Controls (*p* = 0.09). This finding is consistent with our above data in hippocampal lysates. Likewise, acamprosate treatment reduced the number of pERK1/2+ cells in KO mice compared to KO_Controls in the DG (*p* = 0.024). This change was driven by decreases in the number pERK1/2+ neurons in the granule cell layer as evidenced by nuclear co-localization of NeuN in all pERK1/2+ cells in the DG. This suggests that acamprosate can affect neuronal ERK1/2 activation in a manner likely to alter neuronal signal transduction.

### Adult behavior battery comparison of control groups (SAL- vs. CaCl_**2**_-treated mice)

An initial analysis was completed for all behavior paradigms and dependent measures assessed in the adult behavior battery comparing only the two control groups (i.e., SAL- vs. CaCl_2_-treated mice). Complete F statistics are presented in Additional file 1: Tables S1 and S2. No main effects of drug or drug interactions were observed, indicating that CaCl_2_ treatment did not alter the behavior of WT or KO mice compared to those treated with SAL in any test (see Additional file 1: Figure S1). There were significant effects of Genotype, which are further detailed below. Four groups were compared in the final analysis of the behavior battery: (1) WT_Controls (WT + SAL and WT + CaCl_2_ combined), (2) KO_Controls (KO + SAL and KO + CaCl_2_ combined), (3) WT + Acamp, (4) KO + Acamp.

### Elevated zero maze (EZM)

**Fig. 6:**
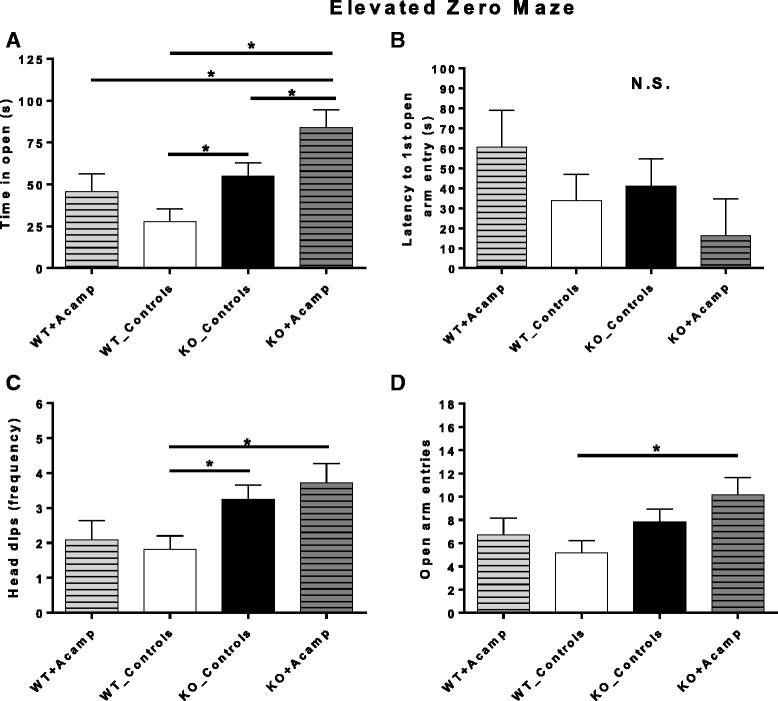
Elevated zero maze (EZM). Wild-type and *Fmr1* KO littermates were treated chronically with either saline or 122.2 mg/kg CaCl_2_ in saline (_Controls; equivalent amount of Ca^2+^ ions as in the 300 mg/kg acamprosate-treated group) or 300 mg/kg acamprosate in saline (+Acamp). The two control groups within each genotype were combined since no main effects of ‘control’ drug or ‘control’ drug interactions were found for any measures in the EZM during initial analysis, which included only saline and CaCl_2_-treated mice from each genotype. Control and Acamp-treated groups were analyzed by two-way ANOVA with pairwise comparisons corrected using FDR (two-tailed) when warranted. There was a significant main effect of gene and drug for time in open (**a**). Pairwise comparisons indicated a baseline genotype increase in time in open in the KO_Controls compared to the WT_Controls. Acamprosate treatment in the KO mice (KO + Acamp) further increased time in open compared to all other groups. No main effects or interactions were noted for Latency to first open arm entry (**b**). There was a significant main effect of gene for head dips (**c**) and transitions (**d**). Both KO groups had more head dips than the WT_Controls group. The KO + Acamp group had more open arm entries than the WT_Controls group. WT_Controls (*n* = 22), WT + Acamp (*n* = 11), KO_Controls (*n* = 20), KO + Acamp (*n* = 11); Data shown are LS mean ± SEM; *p < 0.05 for pairwise comparisons, *N.S.* = not significant

Elevated zero maze was used to assess anxiety behavior in control (SAL- and CaCl_2_-treated) and Acamp-treated *Fmr1* KO and WT mice during a 5-min test. Separate two-way ANOVAs were used to analyze time in open (primary anxiolytic measure), latency to first open arm entry, number of head dips, and number of open arm entries in the EZM (Fig. 6). Pairwise comparison testing using FDR correction (two-tailed) was performed for significant main effects. For time in open, there was a significant main effect of gene (ANOVA, F(1, 60) = 12.41, *p* = 0.001) and drug (ANOVA, F(1, 60) = 6.32, *p* = 0.015; Fig. 6a). Pairwise comparisons showed a significant increase in time in open observed in the open quadrants for the KO_Controls group compared to the WT_Controls group (*p* = 0.031) indicating an observable baseline difference between the two genotypes. In the KO mice, acamprosate treatment further increased time spent in the open quadrants compared to the control-treated KO mice (*p* = 0.049). This increase in the KO + Acamp group was also increased compared to both WT groups (vs. WT_Controls p = 0.001; vs. WT + Acamp *p* = 0.031). For head dip frequency (ANOVA, F(1, 60) = 10.39, *p* = 0.002; Fig. 6c) and number of transitions from dark to light quadrants (ANOVA, F(1, 60) = 5.88, *p* = 0.018; Fig. 6d), there was also a main effect of gene. For number of head dips, the gene main effect was driven by an increase in head dips in both the KO_Controls (*p* = 0.039) and KO + Acamp (*p* = 0.035) groups compared to the WT_Controls. The number of open arm entries was increased in the KO + Acamp mice compared to the WT_Controls (*p* = 0.038) which is consistent with the increase in time spent in the open that was observed for the KO + Acamp group. No significant effects were observed for latency to first open arm entry (Fig. 6b), indicating all mice began exploring the maze at similar times. No other main effects or interactions were noted (see Additional file 1: Table S3 for complete F statistics). Taken together, these data indicate that there was a baseline difference between the KO and WT mice and that acamprosate treatment resulted in an observable behavioral change that is consistent with an anxiolytic effect in only the KO mice.

### Locomotor activity

**Fig. 7:**
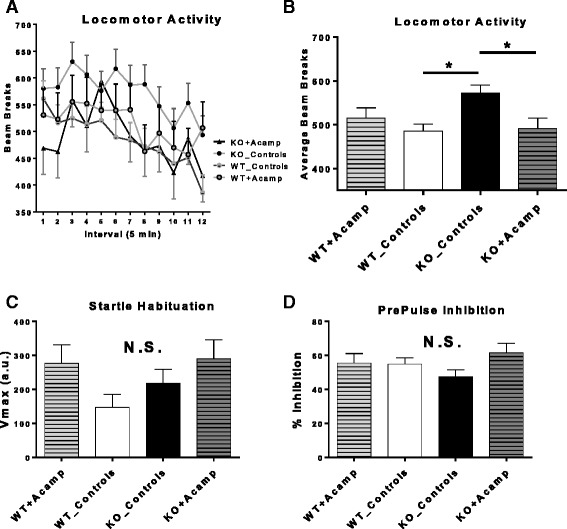
Locomotor activity and acoustic startle habituation/prepulse inhibition. Wild-type and *Fmr1* KO littermates were treated chronically with either saline or 122.2 mg/kg CaCl_2_ (_Controls; equivalent amount of Ca^2+^ ions as in the 300 mg/kg acamprosate treated group) or 300 mg/kg acamprosate (+Acamp). For locomotor activity, a three-way ANOVA with a repeated factor of interval (auto regressive (AR) (1)) revealed main effects of interval and a gene×drug interaction for beam breaks during a 60-min open field test. Panel **a** shows number of beam breaks at each 5-min interval, however, since there was no interaction of interval, pairwise comparisons were performed on beam break data collapsed across time (**b**). Pairwise comparisons corrected using FDR (two-tailed) demonstrated KO_Controls accumulated more beam breaks than WT_Controls, indicating a baseline increase in locomotor behavior in the KO mice. The KO + Acamp mice had reduced beam breaks compared to KO_Controls, indicating a significant effect of acamprosate treatment in the KO mice. No differences between control treatment and acamprosate treatment were evident in the WT mice. In the startle habituation paradigm, a three-way repeated measures ANOVA (AR (1)) for V_max_ revealed a main effect of drug. Pairwise comparisons did not reveal any significant group differences that were maintained following FDR correction (two-tailed) (**c**). For % inhibition during PPI trials, a three-way mixed factor ANOVA with gene and drug as between factors and trial type (PPI73, PPI77, PPI82: PPIxx) as a within factor was used but the omnibus ANOVA did not reveal any significant effects (**d**). For locomotor: WT_Controls (*n* = 24), WT + Acamp (*n* = 11), KO_Controls (*n* = 20), KO + Acamp (*n* = 11). For Habituation: WT_Controls (*n* = 22), WT + Acamp (*n* = 11), KO_Controls (*n* = 20), KO + Acamp (*n* = 11). For % PPI: WT_Controls (*n* = 23), WT + Acamp (*n* = 11), KO_Controls (*n* = 20), KO + Acamp (*n* = 11). Data shown are LS mean ± SEM; *p < 0.05, †*p* < 0.1; *N.S.* = not significant

A three-way repeated measures ANOVA (auto regressive (AR) (1)) for number of beam breaks revealed main effects of interval (ANOVA, F(11, 646) =2.41, *p* = 0.006) and a significant gene×drug interaction (ANOVA, F(1, 114) = 7.06, *p* = 0.009) during the 60-min test (Additional file 1: Table S4). Since there were no interactions with interval (Fig. 7a), FDR-corrected pairwise comparisons (two-tailed) were performed on data collapsed across time (Fig. 7b). There was a significant baseline increase in beam breaks in the KO_Controls group compared to the WT_Controls group (*p* = 0.003). Acamprosate treatment in the KO mice reduced this increase compared to KO_Control mice (*p* = 0.023) such that there was no difference between WT_Controls and KO + Acamp mice (*p* = 0.84). These data indicate that there was a significant baseline difference between the KO and WT mice and that acamprosate treatment normalized open field behavior in the KO mice.

### Novel object recognition (NOR)

Separate two-way ANOVAs (Additional file 1: Table S3) were used to analyze test phase total object attention time and test phase discrimination index (DI) in a short-term object recognition test [76]. During the test phase of NOR, there were no group differences between the total time the mice paid attention to the two objects with the average time being 46.46 ± 3.4 s for WT_Controls, 46.7 ± 3.5 s for KO_Controls, 33.68 ± 5.5 s for WT + Acamp, and 47.43 ± 4.7 s for KO + Acamp (data not shown). There were no main effects or interactions noted for DI (time with the novel object–time with familiar object/time with the novel object + time with the familiar object), nor were there any significant differences between any individual groups (DI LSmean ± SEM, n): WT_Controls = 0.29 ± 0.04, *n* = 21, WT + Acamp = 0.31 ± 0.07, *n* = 8; KO_Controls = 0.27 ± 0.04, *n* = 20; KO + Acamp = 0.26 ± 0.06, *n* = 11; data not shown. All groups spent more time with the novel object (indicated by a DI greater than zero) which suggests that both KO and WT mice were able to remember the familiar object. These data indicate that there was no observable difference in object recognition memory between the control-treated KO and WT mice in this experiment and therefore no deficit to be corrected by acamprosate treatment.

### Acoustic startle habituation

An acoustic startle habituation protocol was utilized to determine if there were differences between WT and KO mice in startle habituation and to acclimate the mice to the chamber and tones for the PPI test assessed 2 days later. A three-way repeated measures ANOVA (Additional file 1: Table S4; auto regressive (AR) (1)) for V_max_ revealed a main effect of drug (ANOVA, F(1,60) = 4.37, *p* = 0.041). However, pairwise comparisons failed to reach significance with FDR correction, indicating little effects of gene or drug on startle habituation in 5–7-month-old mice (Fig. 7c). These data indicate that there was no difference between control-treated WT or KO mice in this acoustic startle habituation test and therefore no deficit that required correction.

### Prepulse inhibition

PPI has been shown to be impaired in young males with FXS, but enhanced in adult male mice [77]. Although the reasons for these discrepancies are unknown, it is clear that both mice and people lacking FMRP exhibit aberrant sensorimotor gating [77, 78]. PPI is a test of startle reactivity and sensorimotor gating and was the final behavior test assessed in the adult behavior battery. PPI was calculated for each animal at each of the prepulse trial types, and a three-way mixed factor ANOVA with gene and drug as between factors and trial type (PPI73, PPI77, PPI82: PPIxx) as a within factor was used. The omnibus ANOVA did not reveal any main effects or interactions for % PPI (Additional file 1: Table S4). There was a trend for a drug×trial type interaction although not significant. Data are shown collapsed across trial type since no interaction of prepulse was detected (Fig. 7d). No differences were detected between control-treated KO and WT mice or in the groups that received acamprosate, suggesting that all groups were similarly able to inhibit the startle response when a prepulse preceded the startle stimulus.

## Discussion

We have shown that acamprosate treatment improved several deficits in cellular, molecular, and behavioral phenotypes in which control-treated *Fmr1* KO mice were found to have deficits compared to control-treated WT mice (see Table 1). Although deficits in seizure susceptibility were apparent between control-treated WT and KO mice, acamprosate treatment did not attenuate this phenotype. Several tests failed to discriminate between WT and KO mice and therefore the treatment effects of acamprosate could not be adequately assessed in these instances.

### Chronic CaCl_**2**_ treatment does not mimic the treatment effects of acamprosate in *Fmr1* KO mice

Spanagel et al. has suggested that the anti-relapse properties of acamprosate (the calcium salt of N-acetylhomotaurinate) and neuroactivity of the molecule are solely due to calcium rather than N-acetylhomotaurinate since an equimolar concentration of a corresponding sodium salt of N-acetylhomotaurinate produced no reductions in alcohol consumption while calcium chloride at equimolar calcium concentrations produced effects similar to acamprosate [53]. It was also suggested that alcohol dependent patients with high plasma calcium levels following treatment with acamprosate had better treatment responses. Although plasma calcium levels in FXS have not been reported to date, FMRP has been shown to regulate several calcium-binding proteins involved in activity-dependent calcium signaling and has been shown to regulate calcium signaling dynamics during development in the dfmr1 null mutant Drosophila FXS disease model [79–82]. As such, the implications that the effects of acamprosate may be reliant on calcium rather than N-acetylhomotaurinate would have significant implications for the future drug development of acamprosate for the treatment of FXS. In the current study, we found that an equimolar concentration of calcium salt, alone, did not produce any effects that were significantly different from saline-treated mice in any behavior paradigm or in any brain regions assessed for pERK1/2 immunostaining in either WT or KO mice. Furthermore, when a treatment effect of acamprosate was observed in the KO mice (EZM, open field, pERK1/2 immunostaining) we did not observe any acamprosate-like effects in the CaCl_2_ group suggesting that the treatment effects of acamprosate in FXS are not due to calcium. Mann et al. recently conducted a study on calcium plasma levels from alcohol dependent patients and showed that there were no differences between acamprosate and placebo-treated patients and that the effect of calcium plasma concentrations on severe relapse was always non-significant. These results also fail to support the hypothesis that calcium is the active moiety of acamprosate [62]. In the current experiments, it is unlikely that differences in calcium bioavailability or elimination rates are likely to affect our results since Chabernat et al. demonstrated that salts of the N-actylhomotaurinate molecule become totally dissociated in hydrophilic media. Since CaCl_2_ is also a hydrophilic molecule, this suggests that the similar amount of Ca2+ ions in both the acamprosate and CaCl_2_ doses used in our current experiments should result in similar Ca2+ bioavailability and elimination rates [83].

With our behavior data demonstrating no differences between SAL and CaCl_2_ treatment, it is unclear why CaCl_2_ had effects on alcohol-seeking behavior as previously reported; however, it is possible that a CaCl_2_ injection may cause some physical discomfort over and above saline treatment due to stinging or burning at the injection site [84]. The mice in our study were treated once-a-day for 10 days prior to behavior testing whereas the rats in the Spanagel et al. paper were injected only twice within 12 h before ethanol intake was assessed. The pain/discomfort from the CaCl_2_ injection may have been sufficient to prevent alcohol seeking whereas in our study, mice may have acclimated to the CaCl_2_ injection, or alternatively, the behavior assessments we conducted were less severely influenced by pain. Although our studies are not able to explain the outcomes of the Spanagel et al. paper, they do suggest that acamprosate rather than calcium may have treatment utility in FXS.

### Acamprosate attenuated spontaneous cortical UP state duration increases but not AGS seizure susceptibility in juvenile *Fmr1* KO mice

UP states are a spontaneous, oscillatory (0.5–1 Hz), synchronized firing of neocortical neuron networks driven by recurrent excitatory and inhibitory synaptic circuits and provide a readout of the intact functioning of neocortical circuits [85, 86]. The examination of spontaneous cortical UP states in the current experiment found prolonged UP state duration in control-treated KO mice compared to control-treated WT mice as expected. Importantly, acamprosate treatment in the KO mice reduced this exaggerated UP state duration. It is thought that the increase in *Fmr1* KO UP state duration is indicative of altered recurrent excitatory signaling or response to signaling through mGluR5 receptor stimulation, as the increased duration remains in the presence of GABA receptor antagonists and is restored to normal by genetic reduction of mGluR5 in *Fmr1* ^-/y^ mice and by the mGluR5 receptor antagonist, MPEP (2-methyl-6-(2-phenylethynyl)pyridine) [25, 87]. Furthermore, Hays et al. demonstrated that depletion of *Fmr1* in glutamatergic neurons but not GABAergic neurons was sufficient to detect increased UP state duration. Acamprosate is suggested to reduce neuronal hyperexcitability, by potentially acting on both glutamate and GABA systems [55, 88–91]. Future work may clarify the mechanism by which acamprosate improves excessive spontaneous cortical activity in *Fmr1* KO mice and to determine if systemic drug treatment has similar effects in vivo.

It has been suggested that *Fmr1* KO-associated increased duration of UP states may contribute to the increased audiogenic seizure susceptibility of juvenile *Fmr1* KO mice, although this has yet to be directly studied. In the current study, we did not observe any reduction in seizure severity score following 5 days of acamprosate treatment in P21 *Fmr1* KO mice. This effect could indicate that spontaneous UP state duration does not directly contribute to seizure susceptibility following intense auditory stimulation. Many non-cortical brain regions are involved in auditory processing, auditory induced seizure behavior, and have been shown to be altered in the *Fmr1* KO mice. Altered spontaneous UP states may not be a critical determinant in AGS susceptibility in these mice, but may contribute to other aberrant behavior in KO mice [92–94]. It is also possible that systemic administration of acamprosate may not have a similar effect on UP state duration as observed in slice application of the drug. It is also possible that a systemic dose of 500 mg/kg of acamprosate may not result in drug concentrations nearing 200 μM in the brain as was bath applied in the UP state study. Additionally, attenuation of UP state duration in these mice may not be sufficient to abrogate increased seizure susceptibility in the AGS test. More work is needed to fully understand any possible connections between FXS-related UP state dysfunction and seizure susceptibility.

### Systemic acamprosate treatment attenuated excessive ERK1/2 activation in *Fmr1* KO mice under basal conditions

The ERK1/2 signaling cascade plays critical roles in brain development and behavior [28]. In neurons, the ERK1/2 cascade is activated by synaptic activity. In turn, ERK1/2 phosphorylates numerous proteins involved in a diverse number of cellular processes including translational and transcriptional regulation, long-term potentiation and depression, and synaptogenesis [30, 95]. In the brain, critical control over temporal and spatial ERK1/2 regulation (nuclear and cytoplasmic), both activation and deactivation, are required for appropriate behavior, and can contribute to maladaptive behavior and central nervous system (CNS) disorders [96–99]. In the first ERK1/2 study (tissue lysates), we observed a ~20% increase in hippocampal and striatal ERK1/2 activation from SAL-treated *Fmr1* KO mice compared to SAL-treated WT mice. This effect has been observed by others using similar techniques [7, 36, 37]. Chronic acamprosate treatment significantly reduced ERK1/2 activation in lysates from both brain regions assessed in acamprosate-treated KO mice compared to control-treated KO mice, indicating a treatment effect. The hippocampus and striatum data characterize ERK1/2 activity in a variety of cell types and throughout the cells (including cytosolic and nuclear ERK1/2) of the regions dissected. Once ERK1/2 is activated in the cytoplasm, it travels to the nucleus where it can then phosphorylate other target proteins and inhibit or activate transcription of many genes [100]. In the second ERK1/2 experiment, the number of cells expressing activated nuclear ERK1/2 immunoreactivity was found to be reduced by acamprosate treatment in the DG, although a difference between control-treated KO and WT mice was only approaching significance with a corrected one-tailed test. The pERK1/2 positively stained cells in these brains were relatively sparse (with no staining in the striatum) and likely represent only those cells with the highest level of nuclear ERK1/2 activity. Nonetheless, we found that in the DG (where we saw a pERK1/2+ cell reduction in acamprosate treated mice), all pERK1/2+ cells were also NeuN+, suggesting that systemic acamprosate treatment modulated neuronal ERK1/2 activity in a cell type- and region-specific manner. Furthermore, CaCl_2_ treatment did not mimic this effect and was indistinguishable from the KO + SAL mice. To our knowledge, these are the first data to suggest that acamprosate modulates central ERK1/2 signaling in vivo and that this change occurs to some degree in the nucleus.

These data are particularly interesting due to the suspected contribution of altered ERK1/2 signaling in FXS and autism pathophysiology. In human study, ERK1/2 activation kinetics following stimulation with phorbol ester have been demonstrated to be delayed in persons with FXS compared to controls [101]. Excessive basal levels of ERK1/2 activation have been reported in FXS mice and in human FXS post-mortem study [39]. In ASD, ERK1/2 dysregulation has been noted in animal model study [102], genetic study [103–105], and in human post-mortem brain study where enhanced ERK1/2 activation has been reported [106]. We and others have shown increases in basal ERK1/2 activation and rescue with various treatments including other GABA and glutamate modulators. Normalization of delayed ERK1/2 activation kinetics with riluzole treatment (glutamate and GABA modulator) was observed in adults with FXS [75]. Both upstream modulators driving increased ERK1/2 activation and the mechanisms by which acamprosate alters ERK1/2 activity in FXS are unknown. However, we have previously shown that acamprosate reduced plasma APP total and secreted APPα levels (sAPPα) in human subjects with FXS [40]. Since APP can induce ERK1/2 activation in vitro [42], there may be a link between the observed effects of acamprosate on APP and ERK1/2 activation in FXS. Furthermore, ERK1/2 activation is thought to be overactive during alcohol withdrawal and suggested to contribute to alcohol dependence and neuronal hyperexcitability associated with chronic alcohol exposure [107]. These data suggest that overactive ERK1/2 signaling associated with other conditions may be attenuated by acamprosate treatment and that one mechanism of acamprosate treatment for alcohol dependence may involve changes in ERK1/2 activation.

Our data and others suggest that central and peripheral ERK1/2 activity in the blood and brain are responsive to neuroactive compounds (including acamprosate). However, more work is needed to determine if these changes impact behavior in a significant way and to what extent ERK1/2 activity can or should be used as a biomarker in FXS. Currently, ERK1/2 activation alterations are being piloted as a biomarker for treatment response and may help identify certain individuals who may respond better to an ERK1/2-modifying drug. Although reduced ERK1/2 activation is typically viewed as the goal of pharmacological treatment in FXS, ERK1/2 signaling abnormalities in FXS are likely much more complicated. Kim et al. demonstrated that in response to synaptic mGluR stimulation, ERK1/2 phosphorylation is rapidly decreased due to over-activated protein phosphatase 2A activity in *Fmr1* KO synaptoneurosomes, whereas in WT samples the opposite occurs resulting in increased phosphorylation/activation [108]. As such, future work is needed to better understand aberrant ERK1/2 signaling abnormalities in FXS, specifically related to cell type, intracellular location, and circuit dysfunction in both drug naïve mice and following pharmacological treatment. It is also critical to determine to what degree any CNS changes in ERK1/2 activity manifest in the type of blood-biomarker samples used in clinical trials.

### Genotype differences and effects of chronic acamprosate treatment were identified in tests of anxiety and locomotor behavior

In the adult behavior battery, we studied the baseline differences between *Fmr1* KO and WT mice in several behavior paradigms and identified genotype differences (WT_Controls vs. KO_Controls) in the EZM and locomotor activity tests. KO mice spent an increased amount of time in the open quadrants of the EZM, suggesting reduced anxiety (opposite of the human phenotype) and were more active in the open field test (hyperactivity and ADHD symptoms are common in individuals with FXS) [109, 110]. Interpretation of rodent EZM or related elevated plus maze data must take locomotor behavior into consideration, since mice that are hyperactive will tend to spend more time in the open quadrants due to increased locomotion. It is possible that the observed increased time in open that is routinely observed in *Fmr1* KO mice, here and by others, is the result of increased locomotor behavior rather than the result of anxiety or risk-taking behavior, although this finding is difficult to reconcile with the human condition [111]. Although we show that treatment with acamprosate further increased time in the open while also reducing open field locomotor behavior in the KO mice, we are unable to determine if treatment reduced anxiety or exacerbated a preexisting abnormality. Interestingly, acamprosate treatment in rodents has been previously associated with anxiolytic properties. In an amphetamine withdrawal-evoked anxiety rodent model, acamprosate treatment increased time in open in the elevated plus maze without a change in locomotor behavior. Another group found that acamprosate reduced social anxiety in a combination stress/ethanol withdrawal rodent model, further supporting the drug’s utility at alleviating anxiety in a manner pertinent to humans with FXS [112, 113]. Koltunowska et al. suggested that this anxiolytic effect of acamprosate may be due to its effects at mGluR receptors which is thought to be a key player in FXS pathophysiology [6]. In human study, open-label treatment with acamprosate in persons with chronic anxiety resulted in reduced anxiety ratings suggesting that acamprosate may modify anxiety behavior although blinded, controlled studies are required to make an accurate determination in this regard [114]. Although the current *Fmr1* KO mouse anxiety data are difficult to interpret, taken together with previous reports in other rodent models and humans with FXS, acamprosate may have utility as an anxiolytic agent in FXS.

Locomotor behavior is not only useful for ensuring proper interpretation of other rodent behavior tests reliant on the movement of the animal but it can also be used to gage baseline levels of hyperactivity. The increased baseline locomotor behavior in *Fmr1* KO mice observed in the current study is consistent with previous data in KO mice as well as well in persons with FXS [115–117]. The attenuation of increased locomotor activity in KO mice with acamprosate treatment is also consistent with our study of acamprosate treatment in person with FXS in which hyperactivity/ADHD symptoms were improved [41]. However, an important distinction must be made between our mouse data and the data that is gathered in many FXS treatment studies related to ADHD symptoms. Open field behavior does not assess ADHD symptoms, but rather the physical activity and movement of mice in a novel environment. One cannot assume that attentional deficiencies in persons with FXS will be improved simply based on reductions in locomotor behavior in rodents. For future clinical trials, the use of wearable activity trackers may improve the translational value of rodent locomotor behavior improvements in FXS studies.

### Lack of phenotypic differences between control-treated WT and KO mice in several paradigms impedes complete characterization of acamprosate treatment effects

Several experiments did not reveal differences between the control-treated KO and WT mice and subsequently conclusions about the treatment effects of acamprosate could not be made in these instances. These tests included object recognition memory, acoustic startle reactivity, prepulse inhibition of the acoustic startle response, and assessment of dendritic spine morphology. Deficits/differences in *Fmr1* KO mice have been observed in these types of experiments previously, but can be difficult to replicate. The experimental parameters are critical determinants in identifying phenotypic deficits in all rodent models, not just *Fmr1* KO mice [118, 119]. For behavior studies, these can include details such as the age of mice at testing, background strain, maternal genotype, loudness/duration of tones, behavior test order, degree of animal handling, inclusion of a pharmacological treatment, injection/treatment exposure route (gavage, IP, food additive), duration of treatment, age at treatment, environmental enrichment, and housing conditions (barrier vs. conventional housing). Cellular and molecular experiments can also be influenced by many experimental parameters including cellular sub-region analyzed (apical vs. basal dendrite/primary vs. secondary branches), methodology of quantification, antibody used, dissection procedure, previous exposure to behavior testing (can function as environmental enrichment condition), staining/imaging techniques, ex vivo vs. culture systems, method of tissue collection/processing (sacrifice method: anesthesia vs. no anesthesia, delay between disruption of the mice and actual time of tissue collection), age at sacrifice. This list is not meant to be exhaustive but meant to highlight the many details that play a role in types of tests commonly used to decipher positive drug effects in FXS translation drug development. Some parameters are at the discretion of the investigator while others are imposed by equipment available or vivarium constraints. In many instances, it is unclear which parameters specifically lead to a significant difference between WT and *Fmr1* KO mice making it difficult to guarantee a particular method will lead to genotype differences at the outset of a preclinical treatment study. In the current study, it is unclear if the age of the mice at testing had any significant effect on a lack of phenotype in NOR or in the acoustic startle tests between the WT and KO mice. Furthermore, a broader characterization of dendritic spine differences may have revealed genotype differences or drug effects. Nonetheless, extrapolation pertaining to the effects acamprosate may have on cognition, sensory reactivity, and gating in humans can not be made from the current results.

The dose used for the adult behavior battery (300 mg/kg) closely matches the clinical dose based on body surface area calculations (see methods for additional information) however, the half-life of acamprosate has been shown to be species dependent. The half-life of acamprosate in humans is approximately 18–32 h following oral administration with 5–7 days of treatment required to reach steady-state plasma concentrations. In rodent plasma, acamprosate has an elimination half-life of 132 ± 56 min, and in brain this can be as short at 43.33 ± 9.55 min [120]. Therefore, the timing of the behavioral tests (1 h following treatment) was chosen to allow mice to recover from the treatment injection while still assessing behavior prior to drug elimination. Furthermore, chronic administration of acamprosate in rodents has been shown to result in increased extracellular brain concentrations of the drug relative to a single treatment suggesting that repeated administration may be needed to achieve clinical efficacy and supports the chronic treatment paradigm used in the current in vivo tests [121].

## Conclusions

Overall, our experience with acamprosate in the *Fmr1* KO mouse demonstrated several challenges of preclinical drug experiments in this field. First, we were unable to capture significant baseline phenotypic deficits in the *Fmr1* KO mouse model in several behavior domains pertinent to the human syndrome. Potential contributors to this may be differences associated with varying background strains and individual lab features that hinder between-lab reproducibility of phenotypic findings with this model. Despite these issues, we were able to demonstrate engagement of acamprosate with elements of pathophysiology of FXS on behavioral, electrophysiological, and molecular levels. Our work highlights the need for transparency in reporting of preclinical trial results in the FXS field so that positive findings can be interpreted in the context of equivocal findings or findings confounded by the lack of baseline deficits at times. Such complete and clear dissemination of results, positive and potentially negative, can aide the choice of initial human study outcome and pharmacodynamic measures thus working to improve the FXS translational treatment pipeline.

## Additional file

Additional file 1: Supplemental Tables and Figures. **Table S1.** Two-way statistical analyses for two control groups: SAL vs. CaCl_2._ **Table S2.** Three-way statistical analyses for two control groups: SAL vs. CaCl_2._ **Table S3.** Statistical analyses for two-way ANOVAs: controls vs. acamprosate treatment. **Table S4.** Statistical analyses for three-way ANOVAs: controls vs. acamprosate treatment. **Figure S1.** Adult behavior battery: no drug effects noted between the two control groups: SAL vs. CaCl_2_. **Figure S2.** pERK1/2+ immunostaining: no drug effects noted between the two control groups: SAL vs. CaCl_2_. (DOCX 442 kb)

## Abbreviations

Acamp: Acamprosate
ADHD: Attention-deficit hyperactivity disorder
AGS: Audiogenic seizure
AMPA: α-amino-3-hydroxy-5-methyl-4-isoxazolepropionic acid receptor
ANOVA: Analysis of variance
APP: Amyloid precursor protein
AR(1): Auto regressive (1)
ASD: Autism spectrum disorder
BDNF: Brain-derived neurotrophic factor
CaCl_2_: Calcium chloride
CCRF: Cincinnati Children’s Research Foundation
CGI-I: Clinical Global Impressions–Improvement
CNS: Central nervous system
DG: Dentate gyrus
DI: Discrimination index
E/I: Excitatory and inhibitory
ELISA: Enzyme-linked immunosorbent assay
ERK1/2: Extracellular-signal regulated kinase 1/2
EZM: Elevated zero maze
FDA: Food and Drug Administration
FDR: False discovery rate
FMR1: Fragile X mental retardation 1 gene
FMRP: Fragile X mental retardation protein
FXS: Fragile X syndrome
GABA: γ-aminobutyric acid
HIP: Hippocampus
IP: Intraperitoneal
KO: Knock-out
lx: Lux
mGluR5: Metabotropic glutamate receptor 5
MPEP: 2-methyl-6-(2-phenylethynyl)pyridine
MTEP: (3-[(2-methyl-1,3-thiazol-4-yl)ethynyl]pyridine hydrochloride)
N.S.: Not significant
NMDA: N-Methyl-D-aspartate
NOR: Novel object recognition
OD: Optical density
pERK1/2: Phosphorylated extracellular-signal regulated kinase ½
PPI: Prepulse inhibition
ROI: Region of interest
SAL: Saline
sAPPα: Secreted amyloid precursor protein alpha
STR: Striatum
USP: United States Pharmacopeia
VEH: Vehicle
WT: Wild-type
