# Supplementary material for: Acamprosate in a mouse model of fragile X syndrome: modulation of spontaneous cortical activity, ERK1/2 activation, locomotor behavior, and anxiety
Source: J Neurodev Disord. 2017 Jun 12;9:6. doi: 10.1186/s11689-017-9184-y (PMC5467053; doi:10.1186/s11689-017-9184-y)
Supplement: Additional file 1: — Supplemental Tables and Figures. Table S1. Two-way statistical analyses for two control groups: SAL vs. CaCl2. Table S2. Three-way statistical analyses for two control groups: SAL vs. CaCl2. Table S3. Statistical analyses for two-way ANOVAs: controls vs. acamprosate treatment. Table S4. Statistical analyses for three-way ANOVAs: controls vs. acamprosate treatment. Figure S1. Adult behavior battery: no drug effects noted between the two control groups: SAL vs. CaCl2. Figure S2. pERK1/2+ immunostaining: no drug effects noted between the two control groups: SAL vs. CaCl2. (DOCX 442 kb) [file 11689_2017_9184_MOESM1_ESM.docx]

**SUPPLEMENTAL TABLES and FIGURES**

**Table S1. Two-way Statistical Analyses for two control groups: SAL vs. CaCl_2_**

|  | **Gene** | **Drug** | **Gene x Drug** |
| --- | --- | --- | --- |
| **pERK+ cells (IHC)** |  |  |  |
| Dentate gyrus | F(1, 18) = 2.23, p = 0.153 | F(1, 18) = 2.08, p = 0.166 | F(1, 18) = 0.35, p = 0.562 |
| Auditory cortex | F(1, 18) = 0.01, p = 0.912 | F(1, 18) = 1.11, p = 0.306 | F(1, 18) = 0.02, p = 0.887 |
| Visual cortex | F(1, 18) = 0, p = 0.982 | F(1, 18) = 0.75, p = 0.397 | F(1, 18) = 0.02, p = 0.879 |
| **EZM** |  |  |  |
| Time in open | F(1, 38) = 17.23, **p = 0.001*** | F(1,38) = 1.06, p = 0.311 | F(1,38) = 0.26, p = 0.6143 |
| Latency to open | F(1,38) = 0.08, p = 0.7723 | F(1,38) = 1.35, p = 0.253 | F(1,38) = 0.04, p = 0.840 |
| Head Dips | F(1,38) = 9.07, **p = 0.005*** | F(1,38) = 0, p = 0.986 | F(1,38) = 0.01, p = 0.930 |
| Transitions | F(1,38) = 5.3, **p = 0.027*** | F(1,38) = 0.67, p = 0.418 | F(1,38) = 0.01, p = 0.935 |
| **NOR** |  |  |  |
| Test phase total time w/objects | F(1,37) = 0, p = 0.971 | F(1,37) = 0, p = 0.996 | F(1,37) = 0.2, p = 0.654 |
| Test phase DI | F(1,37) = 0.21, p = 0.650 | F(1,37) = 0.02, p = 0.894 | F(1,37) = 0.01, p = 0.934 |

Abbreviations: ERK, extracellular signal-related kinase 1/2; pERK, phosphorylated ERK1/2; Elevated Zero Maze, NOR, Novel Object Recognition; DI, discrimination index. *p < 0.05 and **i**ndicate significant effects or interaction.

**Table S2. Three-way Statistical Analyses for two control groups: SAL vs. CaCl_2_**

| **Locomotor: Average Beam Breaks** |  |
| --- | --- |
| Gene | F(1,69.4) = 10.16 **p = 0.0022*** |
| Drug | F(1,69.4) = 0.75, p = 0.390 |
| Gene x Drug | F(1,69.4) = 0.31, p = 0.579 |
| Interval | F(11,417) = 2.09, **p = 0.020*** |
| Gene x Interval | F(11,417) = 0.55, p = 0.870 |
| Drug x Interval | F(11,417) = 1.38, p = 0.182 |
| Gene x Drug x Interval | F(11,417) = 0.66, p = 0.775 |
| **Acoustic Startle Habituation: Vmax** |  |
| Gene | F(1,38) = 4.71, **p = 0.036*** |
| Drug | F(1,38) = 1.14, p = 0.293 |
| Gene x Drug | F(1,38) = 0.24, p = 0.625 |
| Block | F(4,152) = 2.03, p = 0.093 |
| Gene x Block | F(4,152) = 0.41, p = 0.803 |
| Drug x Block | F(4,152) = 0.40, p = 0.811 |
| Gene x Drug x Block | F(4,152) = 0.89, p = 0.471 |
| **ASR/PPI: % Inhibition** |  |
| Gene | F(1, 39) =1.27, p = 0.267 |
| Drug | F(1, 34.6) =0.26, p = 0.616 |
| Gene x Drug | F(1, 38.4) =2.51, p = 0.123 |
| Trial Type | F(2, 78) =2.32, p = 0.105 |
| Gene x Trial Type | F(2, 78) =0.17, p = 0.846 |
| Drug x Trial Type | F(2, 78) =0.4, p = 0.674 |
| Gene x Drug x Trial Type | F(2, 78) =1.02, p = 0.366 |

ASR-PPI, acoustic startle response/prepulse inhibition; *p < 0.05 and **i**ndicate significant effects or interaction.

**Table S3. Statistical Analyses for two-way ANOVAs: Controls vs. Acamprosate treatment**

|  | **Gene** | **Drug** | **Gene x Drug** |
| --- | --- | --- | --- |
| **UP states:** |  |  |  |
| Duration | F(1, 78) = 4.71, **p = 0.0001*** | F(1, 78) =15.74, **p = 0.0002*** | F(1, 78) =1.11, p = 0.296 |
| Amplitude | F(1, 78) = 0.02, p = 0.896 | F(1, 78) =0.51, p = 0.478 | F(1, 78) =1.67, p = 0.200 |
| Number of events | F(1, 78) = 5.14, **p = 0.026*** | F(1, 78) =3.24, p = 0.076† | F(1, 78) =0.09, p = 0.771 |
| **ERK1/2 Hippocampus:** |  |  |  |
| ERK total | F(1, 20) = 1.31, p = 0.27 | F(1, 20) = 1.45, p = 0.24 | F(1, 20) = 0.00, p = 0.97 |
| pERK/ERK total | F(1, 20) = 6.06, **p = 0.023*** | F(1, 20) = 1.42, p = 0.25 | F(1, 20) = 2.99, p = 0.099† |
| **ERK1/2 Striatum:** |  |  |  |
| ERK total | F(1, 20) = 1.06, p = 0.31 | F(1, 20) = 0.00, p = 0.99 | F(1, 20) = 0.50, p = 0.49 |
| pERK/ERK total | F(1, 20) = 3.38, p = 0.08† | F(1, 20) = 5.89, **p = 0.02*** | F(1, 20) = 0.78, p = 0.39 |
| **pERK+ cells (IHC)** |  |  |  |
| Dentate gyrus | F(1, 30) = 0.29, p = 0.592 | F(1, 30) = 7.59, **p = 0.01*** | F(1, 30) = 1.79, p = 0.191 |
| Auditory cortex | F(1, 30) = 1.37, p = 0.251 | F(1, 30) = 0.62, p = 0.439 | F(1, 30) = 0.71, p = 0.406 |
| Visual cortex | F(1, 30) = 0.07, p = 0.795 | F(1, 30) = 0.97, p = 0.333 | F(1, 30) = 0.02, p = 0.897 |
| **EZM** |  |  |  |
| Time in open | F(1, 60) = 12.41, **p = 0.001*** | F(1, 60) = 6.32, **p = 0.015*** | F(1, 60) = 0.35, p = 0.554 |
| Latency to open | F(1, 60) = 1.33, p = 0.253 | F(1, 60) = 0, p = 0.947 | F(1, 60) = 2.55, p = 0.115 |
| Head Dips | F(1, 60) = 10.39, **p = 0.002*** | F(1, 60) = 0.62, p = 0.434 | F(1, 60) = 0.05, p = 0.831 |
| Transitions | F(1, 60) = 5.88, **p = 0.018*** | F(1, 60) = 2.36, p = 0.130 | F(1, 60) = 0.1, p = 0.757 |
| **NOR** |  |  |  |
| Test phase total time w/objects | F(1, 56) = 2.64, p = 0.111 | F(1, 56) = 1.94, p = 0.169 | F(1, 56) = 2.44, p = 0.124 |
| Test phase DI | F(1, 56) = 0.45, p = 0.503 | F(1, 56) = 0, p = 0.961 | F(1, 56) = 0.06, p = 0.807 |

Abbreviations: ERK, extracellular signal-related kinase1/2; pERK, phosphorylated ERK1/2; EZM, Elevated Zero Maze, NOR, Novel Object Recognition; DI, discrimination index; Fam., familiar. *p < 0.05 and **i**ndicate significant effects or interaction.

**Table S4. Statistical Analyses for three-way ANOVAs: Controls vs. Acamprosate treatment**

| **Golgi Staining: Dendritic Spine Counts** |  |
| --- | --- |
| Gene | F(1, 113) = 1.76, p = 0.188 |
| Drug | F(1, 113) = 0.49, p = 0.485 |
| Gene x Drug | F(1, 113) = 3.75, p = 0.055† |
| Segment | F(5, 460) =87.36, **p = 0.0001*** |
| Gene x Segment | F(5, 460) =1.22, p = 0.297 |
| Drug x Segment | F(5, 460) =1.95, p = 0.085† |
| Gene x Drug x Segment | F(5, 460) =0.55, p = 0.741 |
| **Locomotor: Average Beam Breaks** |  |
| Gene | F(1, 114) =2.33, p = 0.130 |
| Drug | F(1, 114) =1.55, p = 0.217 |
| Gene x Drug | F(1, 114) =7.06, **p = 0.009*** |
| Interval | F(11, 646) =2.41, **p = 0.006*** |
| Gene x Interval | F(11, 646) =0.74, p = 0.696 |
| Drug x Interval | F(11, 646) =0.67, p = 0.767 |
| Gene x Drug x Interval | F(11, 646) =0.68, p = 0.763 |
| **Acoustic Startle Habituation: Vmax** |  |
| Gene | F(1,60) = 0.79, p = 0.376 |
| Drug | F(1,60) = 4.37, **p = 0.041*** |
| Gene x Drug | F(1,60) = 0.36, p = 0.552 |
| Block | F(4,240) = 2.28, p = 0.062 |
| Gene x Block | F(4,240) = 1.45, p = 0.218 |
| Drug x Block | F(4,240) = 0.8, p = 0.527 |
| Gene x Drug x Block | F(4,240) = 1.18, p = 0.130 |
| **ASR/PPI: % Inhibition** |  |
| Gene | F(1, 61) =0.02, p = 0.886 |
| Drug | F(1, 55.3) =2.35, p = 0.131 |
| Gene x Drug | F(1, 59.6) =1.18, p = 0.183 |
| Trial Type | F(2, 122) =2.08, p = 0.130 |
| Gene x Trial Type | F(2, 122) =0.74, p = 0.477 |
| Drug x Trial Type | F(2, 122) =3.01, p = 0.0531† |
| Gene x Drug x Trial Type | F(2, 122) =1.02, p = 0.363 |

ASR-PPI, acoustic startle response/prepulse inhibition; *p < 0.05 and **i**ndicate significant effects or interaction; †p < 0.1 and indicate a trend.

**Figure S1. Adult Behavior Battery: no Drug effects noted between the two control groups: SAL vs. CaCl_2_**

**Figure S2. pERK1/2+ Immunostaining: no Drug effects noted between the two control groups: SAL vs. CaCl_2_**
